# Distinct effects of V617F and exon12-mutated JAK2 expressions on erythropoiesis in a human induced pluripotent stem cell (iPSC)-based model

**DOI:** 10.1038/s41598-021-83895-6

**Published:** 2021-03-04

**Authors:** Nungruthai Nilsri, Panchalee Jangprasert, Jaturawat Pawinwongchai, Nipan Israsena, Ponlapat Rojnuckarin

**Affiliations:** 1 Doctor of Philosophy Program in Medical Sciences, Faculty of Medicine, Chulalongkorn University, Bangkok, Thailand; 2 Department of Medical Technology, Faculty of Allied Health Sciences, Naresuan University, Phitsanulok, Thailand; 3 Interdisciplinary Program of Biomedical Sciences, Faculty of Medicine, Chulalongkorn University, Bangkok, Thailand; 4 Faculty of Medical Technology, Rangsit University, Pathum Thani, Thailand; 5 Stem Cell and Cell Therapy Research Unit, Faculty of Medicine, Chulalongkorn University, Bangkok, Thailand; 6 Research Unit in Translational Hematology, Division of Hematology, Department of Medicine, Faculty of Medicine, Chulalongkorn University and King Chulalongkorn Memorial Hospital, Bangkok, 10330 Thailand

**Keywords:** Haematological diseases, Cancer genetics, Cell signalling, Stem cells

## Abstract

Activating mutations affecting the JAK-STAT signal transduction is the genetic driver of myeloproliferative neoplasms (MPNs) which comprise polycythemia vera (PV), essential thrombocythemia (ET) and myelofibrosis. The *JAK2*p.V617F mutation can produce both erythrocytosis in PV and thrombocytosis in ET, while *JAK2* exon 12 mutations cause only erythrocytosis. We hypothesized that these two mutations activated different intracellular signals. In this study, the induced pluripotent stem cells (iPSCs) were used to model *JAK2*-mutated MPNs. Normal iPSCs underwent lentiviral transduction to overexpress *JAK2*p.V617F or *JAK2*p.N542_E543del (JAK2exon12) under a doxycycline-inducible system. The modified iPSCs were differentiated into erythroid cells. Compared with JAK2V617F-iPSCs, JAK2exon12-iPSCs yielded more total CD71^+^GlycophorinA^+^ erythroid cells, displayed more mature morphology and expressed more adult hemoglobin after doxycycline induction. Capillary Western immunoassay revealed significantly higher phospho-STAT1 but lower phospho-STAT3 and lower Phospho-AKT in JAK2exon12-iPSCs compared with those of JAK2V617F-iPSCs in response to erythropoietin. Furthermore, interferon alpha and arsenic trioxide were tested on these modified iPSCs to explore their potentials for MPN therapy. Both agents preferentially inhibited proliferation and promoted apoptosis of the iPSCs expressing mutant *JAK2* compared with those without doxycycline induction. In conclusion, the modified iPSC model can be used to investigate the mechanisms and search for new therapy of MPNs.

## Introduction

Myeloproliferative neoplasms (MPNs) are clonal hematopoietic stem cell disorders caused by acquired activating mutations of cytokine signal transduction resulting in excessive cellular proliferation. The *BCR-ABL* fusion gene is the hallmark of chronic myeloid leukemia, while the main pathogenesis of *BCR-ABL*-negative MPNs is over-activation of the JAK/STAT pathway^1,2^. Clinical syndromes of the latter group comprise polycythemia vera (PV) that is characterized by erythrocytosis, essential thrombocythemia (ET) that shows isolated thrombocytosis and primary myelofibrosis (PMF) that is typified by bone marrow fibrosis and splenomegaly from extramedullary hematopoiesis. Clinical consequences of MPNs are increased risks of thromboembolism, excessive bleeding, exhausted hematopoiesis and transformation to acute myeloid leukemia^3^.

Janus kinase 2 (JAK2) protein is the main signal transducer from both erythropoietin (EPO) and thrombopoietin (TPO) receptors. The gene, *JAK2,* is the most common target of driver mutations with the frequencies of approximately 98% in PV and 50–60% in ET and PMF^4^. The mechanisms underlying these diverse clinical manifestations from a single gene mutation remain unclear. Clinical observations revealed that homozygous *JAK2*p.V617F mutation or, less commonly, heterozygous *JAK2* exon12 mutations, e.g. *JAK2*p.N542_E543del, are found in PV, while heterozygous *JAK2*p.V617F mutation was usually detectable in ET^5^. However, the differences in signal activations responsible for these distinct MPN syndromes are still undefined.

Mouse models have been widely used to investigate the pathophysiology underlying human genetic diseases because mice have almost similar set of genes which can be modified for studies. However, there is a limitation as mice do not always demonstrate similar pathological changes as humans^6^. In order to study hematological diseases, human hematopoietic stem cells must be expanded and maintained ex vivo which is a complicated process. Peripheral blood mononuclear cells are as an easier alternative source, but they have a limited lifespan in culture^7,8^.

Human induced pluripotent stem cells (iPSCs) are generated from mature cells via induction by four factors, Oct3/4, Sox2, c-Myc and Klf4. These stem cells can be derived from either normal subjects or patients to be differentiated into various cell types in vitro. The iPSCs are more likely to represent normal hematopoiesis compared with immortalized cancer cell lines. Furthermore, the cells can be genetically modified and greatly expanded^6,9^. Therefore, iPSCs have potentials to be blood disease models which are probably closer to human physiology than cancer cell lines or animals. Moreover, they may become cell sources for transfusion or immunotherapies in the future.

In this study, we reported the generation of two lines of iPSCs haboring doxycycline-inducible JAK2V617F and JAK2 exon 12 mutants using viral transduction. The iPSCs were derived from normal volunteers instead of patients to avoid interfering effects of concomitant genetic and/or epigenetic alterations in MPN patients. The inducible system in the same iPSC line was used to exclude line-to-line variations because the difference between mutant and control in each experiment was only the expression of the mutant *JAK2* genes. They were the same iPSCs that underwent the same transduction and selection processes followed by freezing, thawing and culturing at the same time under the same conditions. Therefore, the controls were completely matched. The erythrocytes generated from modified iPSCs were enumerated and characterized. Furthermore, the differences in signal activation between these two mutated *JAK2* were explored. This will give us deeper insights in the molecular pathogenesis of MPNs. Subsequently, the mutated iPSCs can be used to screen for the drugs that preferentially affected neoplastic cells more than normal cells. This may lead to novel targeted therapy for MPNs.

## Results

### Generation and characterization of the genetically-modified iPSCs

**Figure 1:**
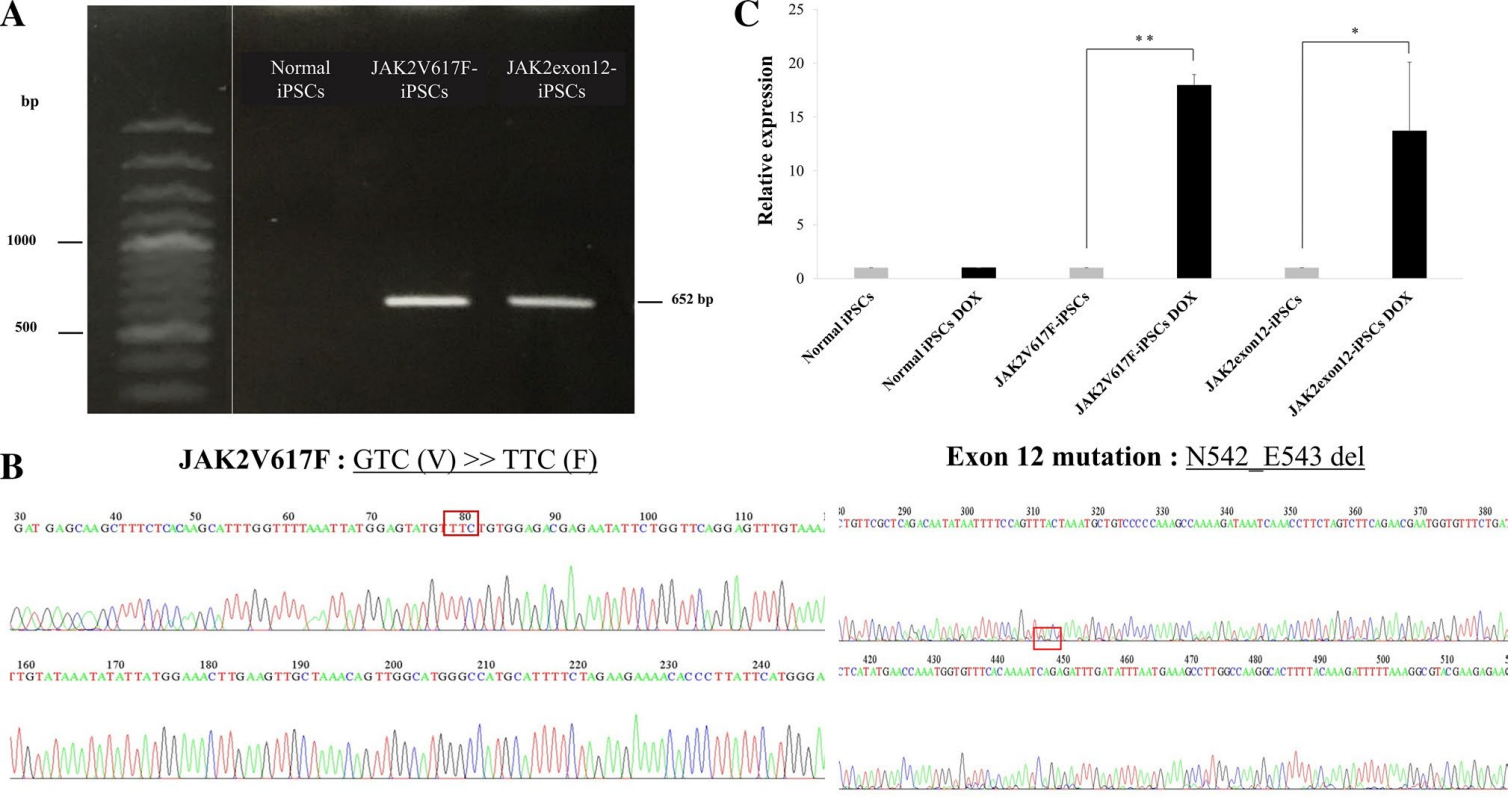
Verification of *JAK2* gene mutations and expression in the modified induced pluripotent stem cells (iPSCs). (**A**) Conventional polymerase chain reaction (PCR) using transgene-specific primers showed exogenous *JAK2* genes in the two modified iPSC lines. The normal iPSC line was used as a negative control. The full gel is presented in the Supplementary Fig. S1A. (**B**) DNA sequencing confirmed the point mutation p.V617F in exon 14 and the p.N542_E543del in exon 12 of *JAK2* gene in the respective iPSC lines. (**C**) Exogenous *JAK2* gene expression levels in iPSCs after transfection comparing normal, JAK2V617F and JAK2 exon 12 mutation with and without doxycycline (DOX) induction for 24 h and analyzed by real-time quantitative RT-PCR. Data are presented as means ± standard deviations (SD) from three independent experiments. The asterisks (*) and (**) denoted *p* < 0.05 and *p* < 0.01.

A normal human iPSC line was modified by overexpressing two types of hyperactive *JAK2* gene mutations (JAK2V617F-iPSCs and JAK2exon12-iPSCs) by using viral transduction. The system used Tet-One inducible expression, in which the inserted gene functioned under the doxycycline control. The modified iPSCs were tested for *JAK2* gene insertion by conventional polymerase chain reaction (PCR) using specific primers to *JAK2*-mutated vectors. Only modified iPSCs demonstrated the PCR products representing an inserted *JAK2* gene, whereas they were absent in normal iPSCs (Fig. 1A). The DNA sequencing confirmed the *JAK2*p.V617F mutation, which was a change from GTC (Valine) to TTC (Phenylalanine) in exon 14, and a deletion at the position N542_E543 of *JAK2* gene in the exon 12 mutation line (Fig. 1B).

The selected iPSCs were determined for the efficiency of doxycycline inducible system by evaluating *JAK2* gene expression. After culturing normal iPSCs and modified iPSCs with and without doxycycline for 24 h, JAK2V617F-iPSCs and JAK2exon12-iPSCs expressed the higher levels of *JAK2* gene after doxycycline exposure at approximately 17.95 ± 1.0 folds (*p* = 0.008) and 13.7 ± 6.4 folds (*p* = 0.034), respectively, when compared with cells in the absence of doxycycline (Fig. 1C).

**Figure 2:**
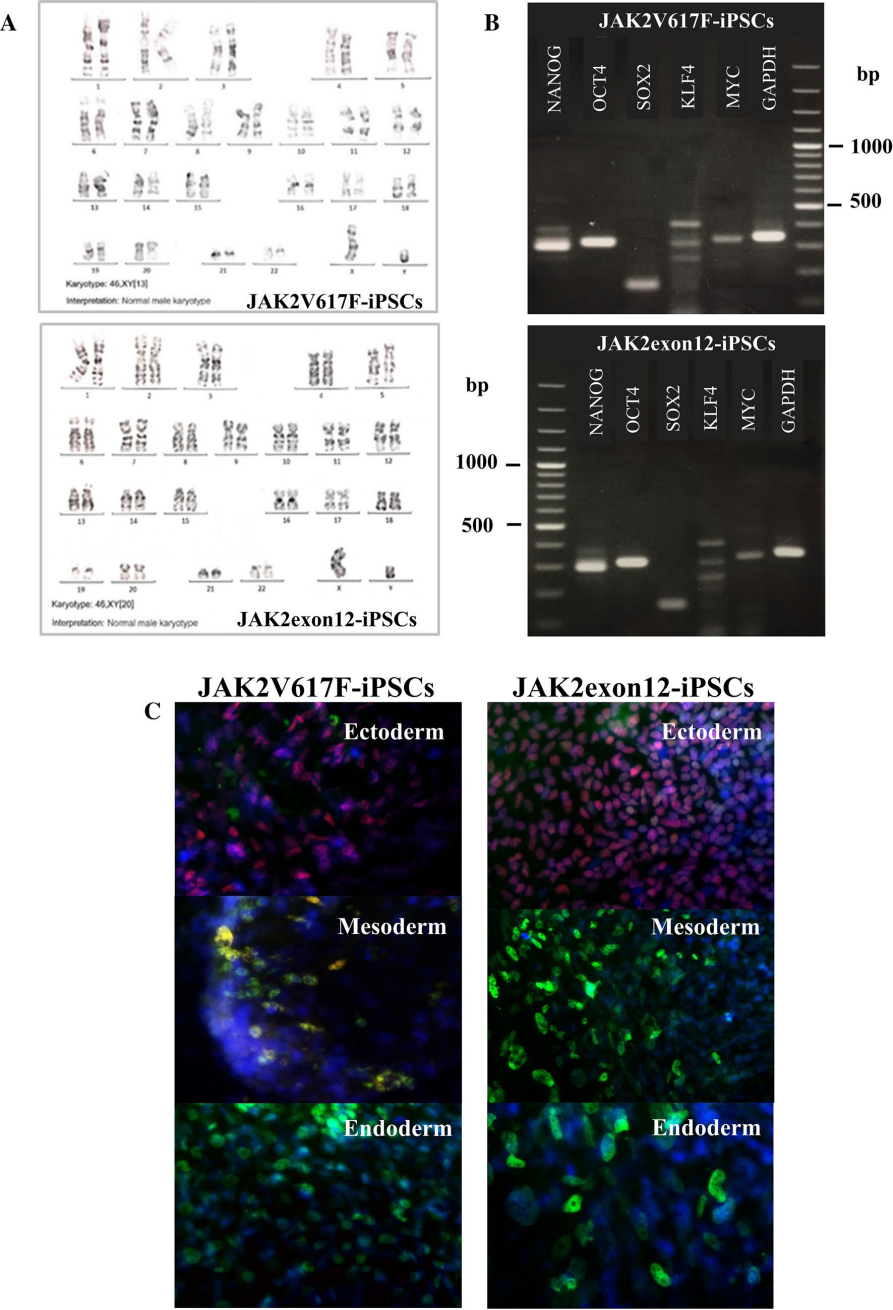
Characteristics of modified induced pluripotent stem cells (iPSCs). (**A**) Karyotyping of the genetically modified iPSCs: JAK2V617F-iPSCs and JAK2N542_E543del-iPSCs (JAK2exon12-iPSCs). (**B**) The expression of *NANOG, OCT4, SOX2, KLF4* and *MYC* of JAK2V617F-iPSCs and JAK2exon12-iPSCs using reverse transcriptase polymerase chain reaction (RT-PCR). The full gels are presented in the Supplementary Fig. S1B. (**C**) Immunofluorescence of differentiated cells from JAK2V617F-iPSCs and JAK2exon12-iPSCs. Embryoid bodies were transferred onto 0.1% gelatin coverslips and cultured for 14 days for differentiation. Cells were stained with antibodies specific to ectoderm (red and green), mesoderm (green), endoderm (green) layers and DAPI (blue) for nuclei (×400 magnification). The images were captured by Axio Observer fluorescence microscopy.

After transduction, JAK2V617F-iPSCs and JAK2exon12-iPSCs demonstrated normal karyotypes in both numbers (46XY) and overall structures of chromosomes (Fig. 2A). Modified iPSC lines showed the RNA expression of five pluripotency genes that were *NANOG, OCT4, SOX2, KLF4* and *MYC* (Fig. 2B). The germ layers from embryoid body differentiation showed positive markers for ectoderm, mesoderm and endoderm by immunofluorescence (Fig. 2C).

### Erythroid cell differentiation

**Figure 3:**
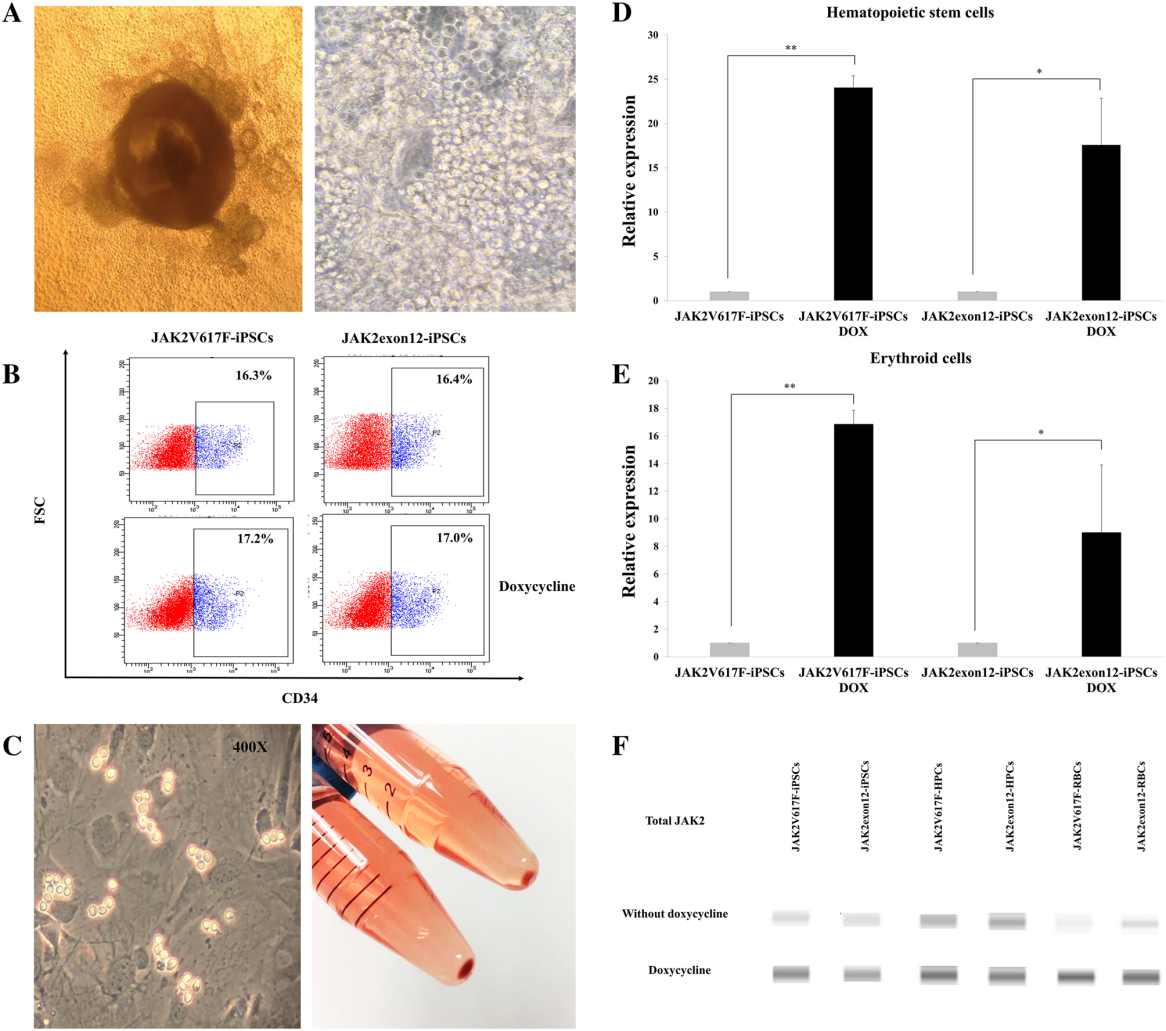
Erythroid cell differentiation from modified induced pluripotent stem cells (iPSCs) via ES-Sacs. (**A**) ES-derived sacs containing hematopoietic progenitor cells were generated from modified iPSCs on day 14 at ×100 and ×400 magnifications. (**B**) The percentages of CD34^+^ cells derived from JAK2V617F-iPSCs and JAK2exon12-iPSCs were similar with or without doxycycline. (**C**) Erythroid cells in a culture plate and red blood cell pellets after centrifugation. (**D**) Expression mRNA levels of exogenous *JAK2* in hematopoietic progenitor cells determined by real-time quantitative RT-PCR. (**E**) Expression mRNA levels of exogenous *JAK2* in erythroid cells. (**F**) The capillary Western immunoassay showed total JAK2 proteins from JAK2V617F-iPSCs and JAK2exon12-iPSCs at the induced pluripotent stem cell, hematopoietic progenitor cell (HPC) and erythroid cell (RBC) stages in the conditions with vs. without doxycycline. Each band was electrophoresed in a separate capillary tube. The full blot is presented in the Supplementary Fig. S2A.

Normal and modified iPSCs that were co-cultured with irradiated C3H10T1/2 feeder cells showed sac-like structures on day 14 of differentiation (Fig. 3A). Hematopoietic progenitor cells from ES-Sacs were collected, passed through a 40-micron cell strainer. The percentages of iPSC-derived CD34^+^ cells from JAK2V617F-iPSCs and JAK2exon12-iPSCs representing hematopoietic stem cells from ES-sacs were all approximately 16–17% with or without doxycycline (Fig. 3B). Hematopoietic stem cells induced by the ES-Sac method were transferred onto fresh feeder cells and then cultured for 15 days. The cells were obtained on day 15 after the initiation of hematopoietic cell culture derived from ES sacs. At that time, round floating cells appeared in culture supernatant (Fig. 3C). After centrifugation, cell pellets showed the red color suggesting the presence of hemoglobin (Fig. 3C).

### The expression of *JAK2* transgenes and protein after doxycycline induction

At the stage of hematopoietic progenitor cells, JAK2V617F-iPSCs and JAK2exon12-iPSCs after induction with doxycycline expressed *JAK2* mRNA at approximately 24.1 ± 1.3 folds (*p* value = 0.002) and 17.6 ± 5.2 folds (*p* value = 0.046), respectively (Fig. 3D). At the erythroid stage, the expression levels were 16.9 ± 1.0 folds (*p* value = 0.003) and 9.0 ± 4.9 folds (*p* value = 0.049) in JAK2V617F-iPSCs and JAK2exon12-iPSCs, respectively (Fig. 3E). Therefore, both hematopoietic progenitor cells and erythroid cells sustained the high levels of *JAK2* transgene expression.

The JAK2 protein expression was also determined by capillary Western immunoassay. JAK2V617F-iPSCs and JAK2exon12-iPSCs expressed higher levels of total JAK2 proteins after doxycycline exposure at approximately 5 and 3 folds, respectively. In addition, JAK2V617F-iPSCs and JAK2exon12-iPSCs at the hematopoietic progenitor cell stage showed the elevations in total JAK2 protein levels of approximately 3 and 1.5 folds, respectively. At the erythroid stage, the JAK2 protein increases were approximately 13 and 9 folds, respectively, compared with cells in the absence of doxycycline (Fig. 3F).

### Characterization of erythroid cells derived from the modified iPSCs

**Figure 4:**
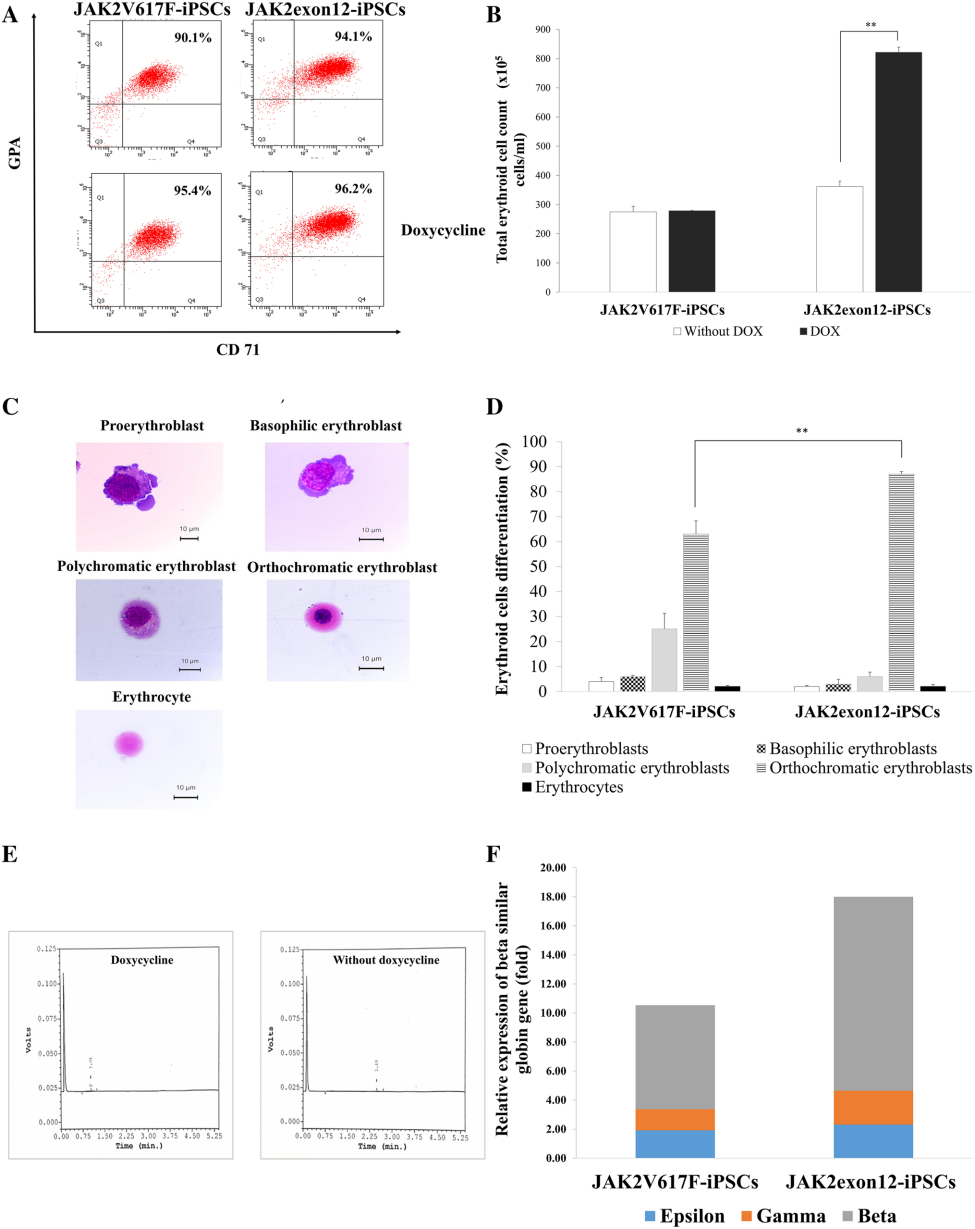
Characteristics of modified induced pluripotent stem cells (iPSC)-derived erythroid cells. (**A**) Flow cytometry of erythroid-specific surface molecules on iPSC-derived erythroid cells without vs. with doxycycline. Cells were stained with PE-conjugated anti-human CD71 and FITC-conjugated anti-human Glycophorin A (GPA) antibodies. (**B**) Total CD71^+^GPA^+^ erythroid cell numbers from erythroid differentiation via ES-Sacs with vs. without doxycycline. (**C**) Five stages of erythroid series were classified by Wright-Giemsa stain consisting of proerythroblasts, basophilic erythroblasts, polychromatic erythroblasts, orthochromatic erythroblasts and erythrocytes. The images were captured by Leica DM 1000 microscopy using LAS49 software and the scale bars represented 10 µm for all panels. (**D**) The percentage of erythroid cell differentiation stages. (**E**) Chromatograms from the ion exchange high performance liquid chromatography (HPLC) Bio-Rad Variant II showed mainly embryonic hemoglobin in modified iPSC-derived erythroid cells with and without doxycycline. (**F**) The relative expression of beta-similar globin genes which were epsilon, gamma and beta in iPSC-derived erythroid cells comparing overexpression of JAK2V617F vs. JAK2 exon 12 mutants and analyzed by real-time quantitative RT-PCR. Data were presented as mean ± SD from three independent experiments. The asterisks (**) denoted *p* < 0.01.

Erythroid cells which were harvested from JAK2V617F-iPSCs and JAK2exon12-iPSCs in the condition without doxycycline on day 29 of differentiation yielded CD71/Glycophorin A (GPA) positivity at approximately 90.1% and 94.1% by flow cytometry, respectively. Cells after doxycycline incubation dually expressed CD71/GPA at 95.4% and 96.2% in JAK2V617F-iPSCs and JAK2exon12-iPSCs, respectively (Fig. 4A).

Regarding the total numbers of erythroid cells, JAK2V617F-iPSCs without vs. with doxycycline produced 275 ± 19.0 × 10^5^ cells/ml vs. 279 ± 1.4 × 10^5^ cells/ml, respectively, while JAK2exon12-iPSCs showed a significant increase in the number of erythroid cells upon stimulation with doxycycline from 362 ± 18.3 × 10^5^ cells/ml (without doxycycline) to 822 ± 17.2 × 10^5^ cells/ml (*p* = 0.007) (Fig. 4B). Only iPSCs expression JAK2 exon 12 mutation showed increase in erythroid cell number and differentiation after doxycycline induction. On the other hand, overexpression of wild-type JAK2 did not show significant increases in the erythroid cell number (Supplementary Fig. S3).

The experiments in the absence and a lower concentration (0 and 2.5 U/ml) of EPO were performed. They showed that the effects of mutant *JAK2* overexpression under these conditions were not prominent as those at 5 IU/ml (Supplementary Fig. S4).

### Morphology of iPSC-derived erythroid cells

During the 29 days of culture, cells from modified iPSCs demonstrated erythroid morphology by Wright-Giemsa staining. The percentage of proerythroblasts, basophilic erythroblasts, polychromatic erythroblasts, orthochromatic erythroblasts and erythrocytes from in vitro erythroid differentiation were demonstrated in Fig. 4C and the most prevalent cells in cultures were orthochromatic erythroblasts. JAK2exon12-iPSCs after doxycycline induction showed a significant increase in the subpopulation of more mature orthochromatic erythroblasts when compared with JAK2V6 17F-iPSCs (*p* = 0.007) (Fig. 4D).

### Hemoglobin analysis

Chromatograms from the Bio-Rad Variant demonstrated the peaks of embryonic hemoglobin at the retention time approximately 0.15 min from iPSC-derived red blood cells with or without doxycycline (Fig. 4E).

Globin gene expression was also evaluated by real-time quantitative RT-PCR. After stimulation with doxycycline, JAK2exon12-iPSCs derived erythroid cells showed a significant increase in the beta globin mRNA expression when compared with JAK2V617F-iPSCs (13.35 ± 0.75 folds vs. 7.16 ± 0.62 folds, respectively, *p* = 0.018) (Fig. 4F).

### JAK/STAT signal transduction

**Figure 5:**
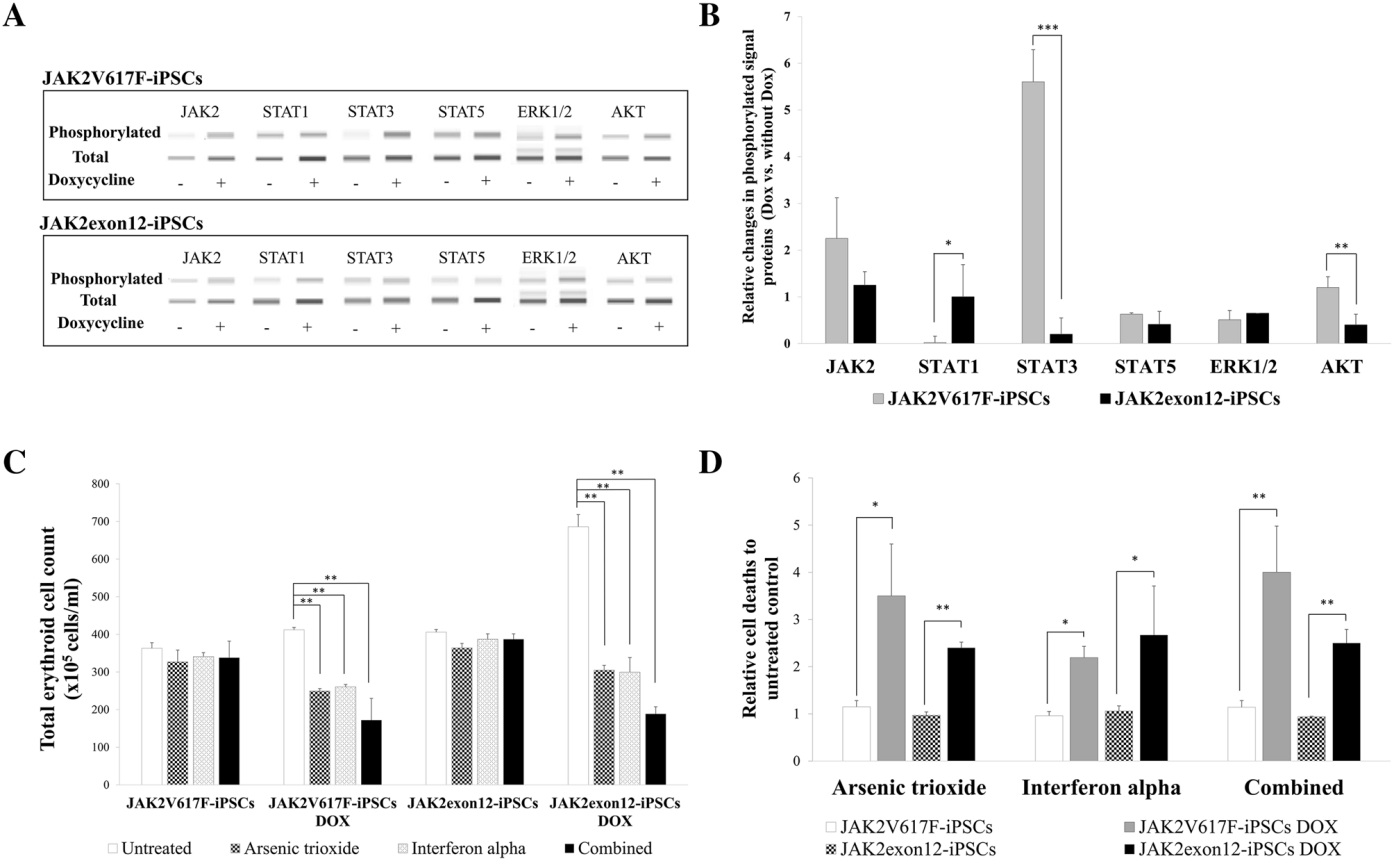
Signal transduction and drug treatment of modified induced pluripotent stem cells (iPSCs). (**A**) The capillary Western immunoassay of phosphorylated and total signaling proteins which were JAK2, STAT1, STAT3, STAT5, ERK1/2 and AKT in the absent and presence of doxycycline in JAK2V617F-iPSCs and JAK2exon12-iPSCs. Each band was electrophoresed in a separate capillary tube. The full blot is presented in the Supplementary Fig. S2B. (**B**) The relative changes of phosphorylated signaling proteins after doxycycline induction in JAK2V617F-iPSCs and JAK2exon12-iPSCs compared with those without doxycycline. The levels of phosphoproteins were corrected by the amounts of respective total proteins. (**C**) Erythroid cell numbers after incubations without (untreated) vs. with arsenic trioxide, interferon alpha and the combination of both drugs. (**D**) The relative increases in numbers of apoptotic cells in modified iPSCs in the presence of arsenic trioxide, interferon alpha and the combination of both drugs. Data were presented as mean ± SD from three independent experiments. The asterisks (*), (**) and (***) denoted *p* < 0.05, *p* < 0.01 and *p* < 0.001, respectively.

Hematopoietic progenitor cells from ES-Sacs were harvested and cultured with a hematopoietic cell differentiation medium supplemented with 5 IU/ml EPO, 50 ng/ml TPO and 50 ng/ml SCF for 24 h. Stimulated cells were collected and then subjected to capillary Western assays for the cell signaling which was composed of JAK2, STAT1, STAT3, STAT5, ERK1/2 and AKT in both native and phosphorylated forms (Fig. 5A). The relative changes in phosphorylated JAK2 proteins in the presence of doxycycline in JAK2V617F-iPSCs and JAK2exon12-iPSCs were 2.50 ± 0.87 and 1.25 ± 0.29 folds, respectively.

JAK2V617F-iPSCs showed significant increases in the relative changes of phospho-STAT3 and phospho-AKT proteins when compared with JAK2exon12-iPSCs at 5.60 ± 0.69 vs. 0.20 ± 0.35 (*p* value < 0.001) and 1.20 ± 0.23 vs. 0.40 ± 0.23 (*p* value = 0.008), respectively. Moreover, the phospho-STAT5 signaling protein level in JAK2V617F-iPSCs was higher than that of JAK2exon12-iPSCs at approximately 0.63 ± 0.03 vs. 0.41 ± 0.28, but there was no statistical significance.

On the other hand, JAK2exon12-iPSCs expressed a higher level of phospho-STAT1 and phospho-ERK1/2 (1.00 ± 0.69 and 0.65 ± 0.00) when compared with JAK2V617F-iPSCs (0.02 ± 0.14 and 0.51 ± 0.20). However, only phospho-STAT1 reached a statistical significance (*p* value = 0.022) (Fig. 5B).

### Interferon alpha and arsenic trioxide treatments

Interferon alpha and arsenic trioxide were tested on modified iPSCs with vs. without doxycycline induction to examine the relative sensitivity of cells expressing mutant *JAK2* compared with wild-type cells. The hematopoietic progenitor cells were cultured in the absence or presence of 0.5 µg/ml interferon alpha and/or 250 nM arsenic trioxide. These optimal concentrations were obtained from cultures using various doses of interferon alpha and arsenic trioxide as demonstrated in Supplementary Fig. S5.

The JAK2V617F-iPSCs showed a significant decrease in the number of erythroid cells after treatment with arsenic trioxide, interferon alpha and the combination of both drugs from 412 ± 6.35 × 10^5^ cells/ml (untreated) to 250 ± 6.35, 261 ± 6.35 and 172 ± 57.4 × 10^5^ cells/ml, respectively. Statistical analyses revealed the significant *p*-values of 0.008, 0.010 and 0.002, respectively.

The JAK2exon12-iPSCs displayed a significant decrease in cell numbers from 686 ± 32.33 × 10^5^ cells/ml (untreated) to 305 ± 12.7 × 10^5^ cells/ml with arsenic trioxide (*p* value = 0.003), 300 ± 38.68 × 10^5^ cells/ml with interferon alpha (*p* value = 0.003) and 189 ± 19.05 × 10^5^ cells/ml with interferon alpha plus arsenic trioxide (*p* value = 0.002). Notably, these agents did not affect the cell numbers of modified iPSCs without doxycycline induction (Fig. 5C).

Concerning the relative changes of apoptotic cells when compared with the untreated control, JAK2V617F-iPSCs after treatments with arsenic trioxide, interferon alpha and the combination of both drugs showed the increases of 3.50 ± 2.10, 2.19 ± 0.42 and 4.00 ± 0.98 folds, respectively, while JAK2exon12-iPSCs displayed the relative changes of approximately 2.40 ± 0.57, 2.67 ± 1.04 and 2.50 ± 0.51 folds, respectively. There was no apoptotic rate difference in modified iPSCs after incubation with either one or both drugs without doxycycline stimulation (Fig. 5D).

## Discussion

From our study, the lentivirus-modified iPSCs retained normal karyotypes, stem cell properties and multi-lineage potentials. The modified iPSC-derived red blood cells displayed erythroid morphology by Wright-Giemsa stain, erythroid surface markers by flow cytometry and embryonic hemoglobin expression similar to normal iPSC-derived cells. Interestingly, JAK2exon12-iPSCs significantly enhanced erythroid cell proliferation mimicking the pathophysiology of JAK2 exon 12 mutations in patients with PV. In addition, expression of *JAK2* with an exon 12 mutant resulted in enhanced erythroid differentiation as shown by more mature morphology and higher expression of adult globin as determined by real-time quantitative RT-PCR. In contrast, JAK2V617F-iPSCs did not show a significant increase in erythroid cell proliferation as enumerated by cell counting and differentiation as evaluated by morphology and hemoglobin analysis despite the overexpression of *JAK2* transgene on real-time quantitative RT-PCR assay and JAK2 protein by Western immunoassay. This disparity may be explained by that JAK2V617F expression in our study was more consistent with heterozygous JAK2V617F mutation in essential thrombocythemia (ET) patients because endogenous *JAK2* gene was still present. The polycythemia vera (PV) patients usually carry homozygous JAK2V617F mutation and, less frequently, heterozygous JAK2 exon 12 mutation as modeled in our study^10^. Therefore, modified iPSCs from this study can be used as an experimental model to investigate the molecular pathogenesis of MPN patients and possibly answer the questions why the JAK2V617F and JAK2 exon 12 mutations attributed to different erythroid phenotypes.

To our knowledge, this is the first report comparing effects of JAK2V617F vs. JAK2 exon12 mutants (*JAK2*p.N542_E543del) overexpression on erythroid development from modified iPSCs and exploring their signaling pathways. Notably, JAK2V617F-iPSCs showed significantly higher relative changes of phospho-STAT3 and phospho-AKT signaling proteins, whereas JAK2exon12-iPSCs exhibited higher amounts of phospho-STAT1. These results were consistent with previous studies in human specimens. Bone marrow biopsies of ET patients who had JAK2V617F mutation showed an increase in phospho-STAT3 by immunohistochemical analysis and immunoblotting^11^. Additionally, bone marrow trephine biopsy sections of MPN patients, JAK2V617F mutation was associated with significantly increased levels of phospho-STAT5 and phospho-AKT in hematopoietic cells, which were most prominent in megakaryocytes^12^. Furthermore, cells reprogrammed from heterozygous JAK2V617F patients showed a high level of phospho-STAT5 and displayed TPO-independent formation of megakaryocytic colonies but not EPO-independent erythroid colony^13^. Supporting these data, the study in BaF3/EPOR cells transduced with various types of *JAK2* gene mutations including JAK2V617F, N542_E543del, H538QK539L, K539L and F537_K539delinsL showed that these exon 12 mutations activated the RAS-ERK signaling pathway. The levels of phospho-ERK1 and ERK2 were markedly higher than JAK2V617F mutation and there were variable levels of phospho-ERK in different types of exon 12 mutations^14^. In addition, the STAT1 knockout mice showed reduced bone marrow-derived erythroid colony forming units and less differentiated phenotypes associated with increased apoptosis of early erythroblasts. These data demonstrated that STAT1 played a critical role in the regulation of erythropoiesis^15^. On the other hand, STAT3 is probably a minor signaling molecule for EPO-independent growth but may play an important role in megakaryopoiesis. The stronger effects of the JAK2exon 12 mutation may be from the more prominent STAT1 activation compared with those of the JAK2V617F. Therefore, dissimilar signals may explain the different phenotypes of patients with heterozygous JAK2V617F and those with *JAK2* exon 12 mutations.

In 2013, iPSCs containing heterozygous and homozygous JAK2V617F were generated from MPN patients and studied for molecular mechanisms. However, MPN patient samples usually co-carried other genetic defects including 20q deletion, *ASXL1, FBXO15* and *MATN2*^13^ mutations that can affect iPSC phenotypes. Clonal subpopulation may vary among samples depending on disease progression and treatment processes. Furthermore, different clones of iPSCs may display distinct intracellular signaling and growth potentials. In this study, the iPSC lines with doxycycline-inducible *JAK2* mutations were constructed from normal iPSCs. The in vitro erythrocyte generation was observed comparing between cultures with vs. without doxycycline. Therefore, the phenotype differences were attributed solely to the overexpressed mutated *JAK2* genes without interferences by other genetic and/or epigenetic background.

A possible limitation of our model is that iPSC-derived hematopoietic stem cells (HSCs) might show different properties compared with the marrow-derived HSCs. Mascarenhas et al. revealed that the mouse embryonic HSCs were relatively resistant to JAK2V617F mutation compared with adult HSCs^16^. Consistent with this finding, we found that JAK2V617F expression also showed minor effects on erythropoiesis from human iPSCs. However, our experiments revealed that overexpressing JAK2 with mutated exon 12 in human iPSCs significantly increased erythropoiesis similar to the erythrocytosis phenotype in patients. Therefore, the iPSC model gave us the opportunities to demonstrate that the different effects of mutant JAK2s were correlated with different STAT signals and to explore the drugs that selectively inhibit cells with mutant JAKs. These yielded a deeper insight in the pathogenesis of MPNs and may lead to future therapy.

In the past, potentially new drugs were screened in immortalized cancer cell lines and animal models which cannot always predict efficacy and safety in humans^17^. The iPSCs can be differentiated into disease specific cell types and demonstrate the phenotypes similar to primary cells. Drug screening on these iPSC-derived cells may be helpful for discoveries of novel treatments.

Interferon alpha that signals through the JAK/STAT pathway has been used for the treatments of ET or PV. The mechanisms of action of interferon-alpha have been ascribed to its anti-proliferative, pro-apoptotic, anti-angiogenic, and immunomodulatory effects^18^. Interestingly, interferon can decrease the mutated *JAK2* allele burdens in MPN patients. In addition, the combination with the other drugs may be more efficacious for advanced and transformed diseases^19^. There were reports that interferon alpha preferentially induced JAK2-positive cell apoptosis which was mediated through the p53 or p38MAPK pathways^20,21^. Furthermore, recent data found that the interferon-sensitivity depended on STAT2 activation^22^. Arsenic trioxide is the standard treatment for relapsed acute promyelocytic leukemia (APL) through promoting apoptosis which is involved intracellular glutathione and hydrogen peroxide^23^. Recent researches showed that hematologic malignancies other than APL also responded to combination therapy containing arsenic trioxide. JAK2V617F-UT7 cell lines were generated and revealed the synergistic effects of interferon alpha and arsenic trioxide combination^24^. Arsenic trioxide was shown to induce acute promyelocytic cell (APL) differentiation at low concentrations and apoptosis at high concentrations of over 500 nM partly from the specific degradation of the PML-RARα oncoprotein^25^. The proposed mechanisms of arsenic trioxide in other cancers are the increases in reactive oxygen species (ROS) from mitochondria and/or endoplasmic reticulum causing cellular apoptosis^26^.

According to our experiments, JAK2V617F-iPSCs and JAK2exon12-iPSCs showed a significant decrease in the number of erythroid cells and an increase in apoptotic cells after treatment with arsenic trioxide, interferon alpha and the combined regimen. The additive effect of these two agents was observed in our model. Interestingly, arsenic trioxide and interferon alpha treatments showed the specific effects on mutated iPSCs but did not in the condition without doxycycline induction. This disease model of overexpressing JAK2V617F and JAK2 exon 12 mutants suggests the potential roles of interferon alpha and arsenic trioxide in therapy of MPN patients. To explore whether interferon alpha and/or arsenic trioxide also affected other lineages, the experiments were performed during the megakaryopoiesis. Similarly, these agents preferentially suppressed the proliferation of mutant JAK2 expressing cells as compared with cultures without doxycycline induction (Supplementary Fig. S6). Therefore, they have a potential to eliminate the malignant clone. In the future, this modified iPSCs can be used to test for other new therapeutic agents.

Derivation of red blood cells from iPSCs may become blood products for transfusion. Genetic engineering can be applied to generate erythrocytes with very rare blood groups without requirement for exceptional donors. The proteome analysis of erythroid cells differentiated from iPSC lines revealed a similar pattern to that of normal adult erythroid cells^27^. However, the challenges of erythroid production are inefficient enucleation, low expression of the adult β hemoglobin and scalable production^28^. From our result, JAK2exon12-iPSCs enhanced red blood cell production with a greater number of the late-stage erythrocytes and produced more adult hemoglobin (α2β2, HbA) at the mRNA level. Overexpression of *JAK2* with exon 12 mutations may be one of the factors to improve the blood cell production for transfusion.

## Conclusions

Our study used the iPSC technology to obtain better understanding of the *JAK2* mutation effects on erythropoiesis. The JAK2 exon 12 mutation strongly promoted erythroid cell proliferation and differentiation correlating with STAT1 and ERK activation. Modified iPSCs provided a model to study the mechanisms of mutated *JAK2*, screen for novel therapeutic agents and possibly offer a potential source for red blood cell transfusion in the future.

## Materials and methods

### Establishing the iPSC lines with *JAK2* gene mutations

A normal human iPSC line was modified by overexpressing two types of hyperactive *JAK2* gene, which were a point mutation in exon 14 (*JAK2*p.V617F) and a small deletion in exon 12 (*JAK2*p.N542_E543del), using viral transduction. The experimental design using human iPSCs was approved by the Institutional Review Board of the Faculty of Medicine at Chulalongkorn University, Bangkok, Thailand (Certificate No. 33/2018) and was conducted in accordance with the Declaration of Helsinki. The iPSCs were derived from skin fibroblasts of a healthy subject after informed consent. The reprogramming process was described in a previous study^29^. The details on iPSC characterization of pluripotency were described in the Supplementary Fig. S7.

Firstly, the wild-type *JAK2*-containing plasmid (Addgene, Cambridge, MA, USA) was altered using the Site-directed mutagenesis kit (Thermo Fisher Scientific, Waltham, MA, USA). The constructed plasmids carrying *JAK2*p.V617F (JAK2V617F) and *JAK2*p.N542_E543del (JAK2exon12) were inserted into the complementary site of pLVX-TetOne-Puro vector by which the mutated *JAK2* were expressed under the Lentiviral Tet-One inducible expression system (Clontech, Palo Alto, CA, USA). For lentiviral production, the recombinant vectors containing either JAK2V617F or JAK2exon12 coding sequences were transfected into 293FT cells. Virus-containing supernatants were incubated with normal iPSCs in the presence of 6 µg/ml polybrene for transduction. Transfected iPSCs were selected by puromycin^30^.

To verify the engineered cell lines, modified iPSCs were harvested and extracted for genomic DNA by using the prepGEM kit (MicroGEM, Aotearoa, New Zealand). The DNA from modified iPSCs was assayed for *JAK2* gene insertions using polymerase chain reaction (PCR) and sequencing. Each tube contained cDNA (100 ng/µl final concentration), 10 µM forward and reverse primer mix specific for exogenous *JAK2* (The primer sequences are listed in Table 1) and GoTaq Master Mixes (Promega, Madison, Wisconsin, USA). The PCR condition was 95 °C initial denaturation for 5 min, followed by 30 cycles of denaturation (98 °C, 40 s), annealing (63 °C, 1 min), and extension (72 °C, 1.30 min). Unmodified iPSCs were used as a negative control. DNA templates were amplified in a T gradient Biometra thermal cycler (Biometra GmbH, Göttingen, Germany). Sanger DNA sequencing was used to confirm *JAK2* gene mutations and analyzed by capillary electrophoresis at Macrogen Inc., Korea.

**Table 1:** The primer sets for exogenous *JAK2*, pluripotency testing of stem cells and hemoglobin expression.

Genes	Forward primers	Reverse primers
Exogenous *JAK2*	CCCTCGTAAAGAATTCATGGGAATGGCC TGCCTTACGATG	TCTTTGCTCGAATACATTTTGG
*NANOG*	ATACCTCAGCCTCCAGCAGA	CAGGACTGGATGTTCTGGGT
*OCT4*	GAAGGTATTCAGCCAAACGC	GTTACAGAACCACACTCGGA
*SOX2*	GGGAAATGGGAGGGGTGCAAAAGAGG	TTGCGTGAGTGTGGATGGGATTGGTG
*KLF4*	ACGATCGTGGCCCCGGAAAAGGACC	TGATTGTAGTGCTTTCTGGCTGGGCTCC
*MYC*	GCGTCCTGGGAAGGGAGATCCGGAGC	TTGAGGGGCATCGTCGCGGGAGGCTG
Epsilon globin	GCCTGTGGAGCAAGATGAAT	GCGGGCTTGAGGTTGT
Gamma globin	TGAGAACTTCAAGCTCCTGGGAAA	TGCAGAATAAAGCCTATCCTTGAA
Beta globin	TACATTTGCTTCTGACACAAC	ACAGATCCCCAAAGGAC

The selected iPSCs were examined for the efficiency of doxycycline inducible system. Cells were cultured in the medium with 0 or 2 µg/ml of doxycycline (DOX, Stemcell Technologies, Vancouver, BC, Canada) and incubated for 24 h before harvesting cell pellets and tested for *JAK2* gene expression by using real-time quantitative RT-PCR. PCR was performed using exogenous *JAK2*-specific primers and the following protocol: 95 °C initial denaturation for 10 min, followed by 40 cycles of denaturation (95 °C, 15 s), annealing (59.5 °C, 30 s), and extension (72 °C, 45 s).

### Characterization of genetically-modified iPSC properties

The genetically-modified iPSCs were required to ensure the normal karyotypes, the pluripotent status and the capacity to differentiate into cells of the three germ layers.

Chromosomal analysis was performed by GTG-banding analysis at the Center for Medical Diagnostic Laboratories, Faculty of Medicine, Chulalongkorn University, Thailand, following the recommendations by the International System for Cytogenetics Nomenclature (ISCN).

The pluripotency of stem cells was evaluated by reverse-transcriptase PCR (RT-PCR) of stem cell markers including *NANOG, OCT4, SOX2, KLF4* and *MYC*. The primer sequences are listed in Table 1^31–34^.

The ability of stem cells to differentiate into endoderm, mesoderm and ectoderm were tested via embryoid body (EB) formation and stained by Human three germ layer 3-color immunocytochemistry kit (R&D Systems, Minneapolis, MN, USA)^35^.

To demonstrate the hematopoietic multipotency, our iPSC-derived hematopoietic progenitors were cultured in the presence of 100 ng/ml human thrombopoietin (TPO), 50 ng/ml human stem cell factor (SCF), and 25 ng/ml heparin for 21 days. The culture finally yielded the mixture of megakaryocytes (by morphology and flow cytometry for CD41/CD42b surface expression) and neutrophils (by morphology) as shown in the Supplementary Fig. S8.

### Reverse transcriptase polymerase chain reaction (RT-PCR) and real-time quantitative RT-PCR

Total RNA from modified iPSCs were extracted using an RNA purification kit (GeneJet kit; Thermo Fisher Scientific, Waltham, MA, USA). Isolated RNA was reverse transcribed using a cDNA synthesis kit (Thermo Fisher Scientific).

Real-time quantitative RT-PCR assay was performed by using Capital qPCR probe mix (Biotechrabbit GmbH, Hennigsdorf, Germany) on 7500 Fast real-time PCR system (Applied Biosystems, Foster City, CA, USA). The relative quantity of each target gene was normalized to glyceraldehyde-3-phosphate dehydrogenase (GAPDH) as a house-keeping gene. Fold changes were calculated by quantifying expression using comparative CT (ΔΔCt) method compared with those of normal unmodified iPSCs. All samples were processed in triplicate.

### Differentiation of iPSCs into erythrocytes

Normal iPSCs and modified iPSCs were differentiated into erythroid cells using the ES-sac method according to Ochi et al.^36^. Cells were dissociated into small pieces (> 100 cells) by collagenase treatment. Small clump of cells were transferred onto irradiated C3H10T1/2 feeder cells and cultured in a hematopoietic cell differentiation medium, Iscove's Modified Dulbecco's Medium (IMDM) supplemented with 10 µg/ml human insulin, 5.5 µg/ml human transferrin, 5 ng/ml sodium selenite, 2 mM l-glutamine, 0.45 mM α-monothioglycerol, 50 µg/ml ascorbic acid and 15% fetal bovine serum (FBS) containing 20 ng/ml recombinant human vascular endothelial growth factor (rhVEGF, R&D Systems) with 0 or 2 µg/ml of doxycycline from the first day of culture.

**Figure 6:**
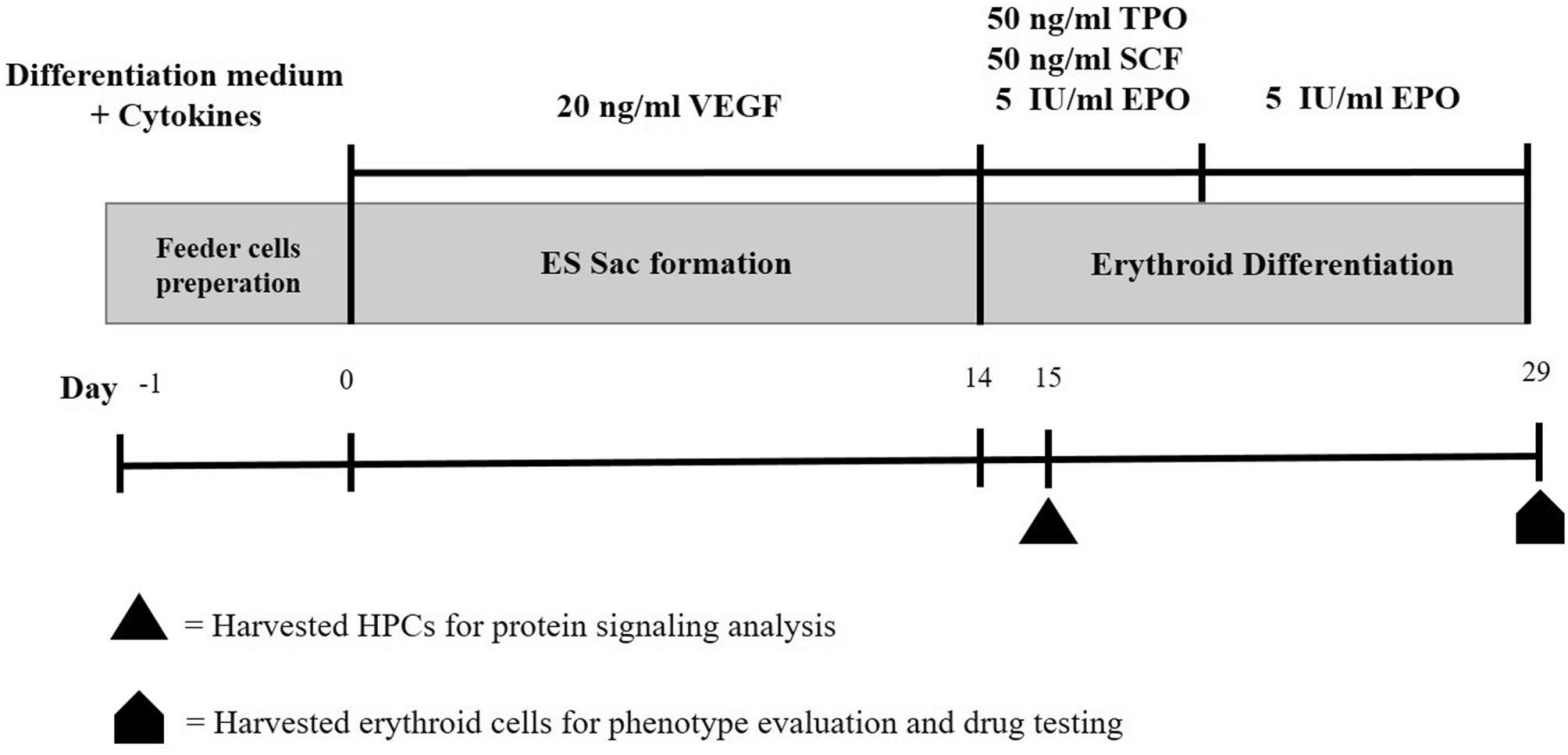
Schematic diagrams of in vitro differentiation protocols for erythrocytes production via ES-Sacs formation. *VEGF* Vascular endothelium growth factor, *TPO* thrombopoietin, *SCF* stem cell factor, *EPO* erythropoietin, *HPCs* hematopoietic progenitor cells.

On day 14 of culture, embryonic stem cell–derived sacs (ES-Sacs) were emerged. Cells from ES-Sacs were gently crushed with a needle and passed through a 40-µm cell strainer which selected a population of hematopoietic progenitor cells (HPCs). The hematopoietic progenitors at 5 × 10^4^ cells/ml were maintained in hematopoietic cell differentiation medium supplemented with 50 ng/ml human thrombopoietin (TPO, R&D Systems), 50 ng/ml human stem cell factor (SCF, R&D Systems) and 5 IU/ml erythropoietin (EPO, Eprex, Janssen Pharmaceutical, Beerse, Belgium) and then transferred onto fresh and irradiated C3H10T1/2 cells in a six-well plate for 6 days. After 6 days, cells were transferred to fresh irradiated C3H10T1/2 cells and cultured in hematopoietic cell differentiation medium supplemented only with 5 IU/ml EPO for another 9 days. Non-adherent cells were harvested and analyzed on day 29 of culture^36^ (Fig. 6).

### Flow cytometry analysis

Erythroid cells on day 29 were incubated with PE-conjugated anti-human CD71 (Clone CY1G4, BioLegend, San Diego, CA, USA) and FITC-conjugated anti-human Glycophorin A (GPA, Clone HI264, BioLegend) at room temperature in the dark for 30 min. Flow cytometry was performed by using BD FACSAria II (Becton Dickinson, Franklin Lakes, NJ, USA). The total erythroid cells were calculated by counting the total numbers of cells and multiplying by the percentages of CD71^+^GPA^+^ cells.

### Morphological analysis

The modified iPSCs on day 29 of culture were classified by morphology into various differentiation stages of the erythroid lineage. Cells were harvested from culture supernatant, mounted on slides by Cellspin I 1–12 (Tharmac GmbH, Wiesbaden, Germany) and stained by Wright-Giemsa with phosphate buffer. Subsequently, erythrocytes were observed under Leica DM 1000 microscopy (Leica, Wetzlar, Germany).

### Hemoglobin typing

The Variant II Beta Thalassemia Short Program utilizing the ion-exchange high-performance liquid chromatography (HPLC) principle (Bio-Rad, California, United States) was applied.

The mRNA of beta globin subtype genes were measured by real time quantitative PCR to determine the expression levels of embryonic hemoglobin (epsilon; ε), fetal hemoglobin (gamma; γ) and adult hemoglobin (beta; β). The primer sequences are listed in Table 1^37–39^.

### JAK/STAT signal transduction

From ES-Sacs formation, the hematopoietic progenitor cells were harvested and transferred onto fresh irradiated C3H10T1/2 cells and then cultured with a hematopoietic cell differentiation medium supplemented with 50 ng/ml TPO, 50 ng/ml SCF and 5 IU/ml EPO for 24 h. Stimulated cells were measured for protein concentrations using Micro BCA protein assay kit (Thermo Fisher Scientific).

Signaling protein analyses were performed on a capillary Western immunoassay system (WES, ProteinSimple, California, USA) using antibodies to JAK2, STAT1, STAT3, STAT5, ERK1/2 and AKT in both native and phosphorylated forms (Cell Signaling Technology, Massachusetts, USA). The chemiluminescent signals were detected and quantitated by Program Compass for SW. The level of each phosphoprotein was corrected by the amount of the respective total signaling protein and later calculated for the fold changes comparing with vs. without doxycycline induction.

### Effects of drugs on erythroid development from modified iPSCs

The hematopoietic progenitor cells generated from modified iPSCs at 5 × 10^4^ cells/ml were cultured in the absence or presence of drugs which had potentials to treat MPN patients, 0.5 µg/ml interferon alpha-2a (Roche, New Jersey, USA) and/or 250 nM arsenic trioxide (M&B, London, United Kingdom)^40,41^.

The yields of total erythroid cells were enumerated as above. The percentage and relative changes of cell deaths were determined using FITC-conjugated anti-human Glycophorin A (Clone HI264, BioLegend) and propidium iodide (#421301, BioLegend). The results were compared between with vs. without doxycycline to determine the differential effects on mutated vs. normal cells, respectively.

### Statistical analysis

Statistical analyses were performed using the SPSS software (version 22.0). All the continuous variables were expressed as means ± standard deviations (SD). The statistical differences between groups (doxycycline induction vs. no doxycycline induction of mutated *JAK2* transgene expression) were determined using the paired T-test. In addition, one way ANOVA and independent-sample T-test were also used to detect statistical significances. The probability (P) values of less than 0.05 were considered statistically significant.

## Supplementary Information

Supplementary Information.
