# Supplementary material for: Distinct effects of V617F and exon12-mutated JAK2 expressions on erythropoiesis in a human induced pluripotent stem cell (iPSC)-based model
Source: Sci Rep. 2021 Mar 4;11:5255. doi: 10.1038/s41598-021-83895-6 (PMC7933160; doi:10.1038/s41598-021-83895-6)
Supplement: Supplementary file 1 — Supplementary Information. [file 41598_2021_83895_MOESM1_ESM.docx]

**Distinct effects of V617F and exon12-mutated JAK2 expressions on erythropoiesis in a human induced pluripotent stem cell (iPSC)-based model**

Nungruthai Nilsri^1,2^, Panchalee Jangprasert^3^, Jaturawat Pawinwongchai^4^, Nipan Israsena^5^, Ponlapat Rojnuckarin^6, #^

^1^Doctor of Philosophy Program in Medical Sciences, Faculty of Medicine, Chulalongkorn University, Bangkok, Thailand

^2^Department of Medical Technology, Faculty of Allied Health Sciences, Naresuan University, Phitsanulok, Thailand

^3^Interdisciplinary Program of Biomedical Sciences, Faculty of Medicine, Chulalongkorn University, Bangkok, Thailand

^4^ Faculty of Medical Technology, Rangsit University, Pathum Thani, Thailand

^5^Stem cell and Cell Therapy Research Unit, Faculty of Medicine, Chulalongkorn University, Bangkok, Thailand

^6^Research Unit in Translational Hematology, Division of Hematology, Department of Medicine, Faculty of Medicine, Chulalongkorn University and King Chulalongkorn Memorial Hospital, Bangkok, Thailand

**Supplementary Figures**


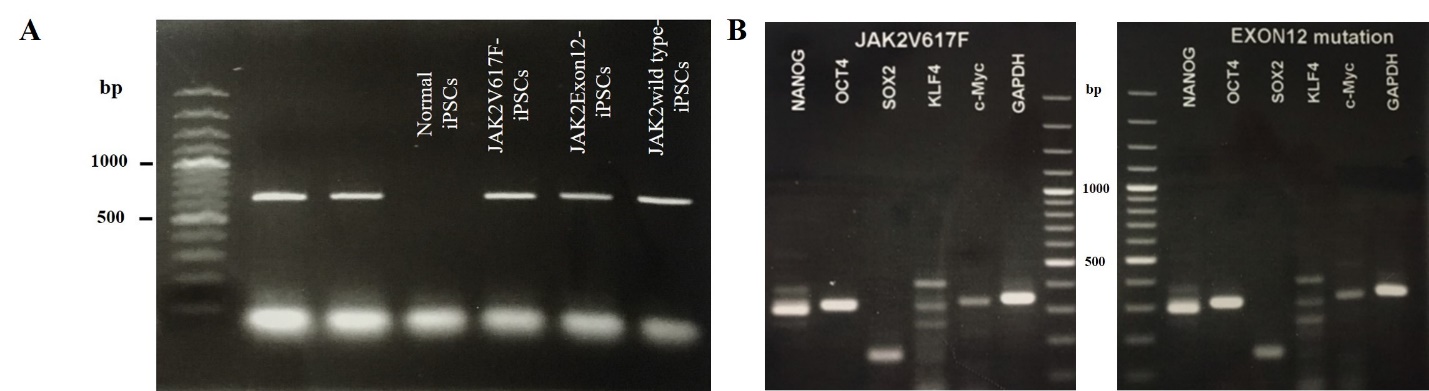


**Supplementary Figure S1.** The full-length gels A) The exogenous *JAK2* gene expression: Normal and modified iPSCs were extracted for genomic DNA and assayed for the exogenous *JAK2* gene using polymerase chain reaction (PCR). B) The pluripotency gene expression of the two modified iPSC lines: The harvested cell pellets were evaluated by reverse-transcriptase PCR using stem cell marker primers including *NANOG, OCT4, SOX2, KLF4* and *MYC.*


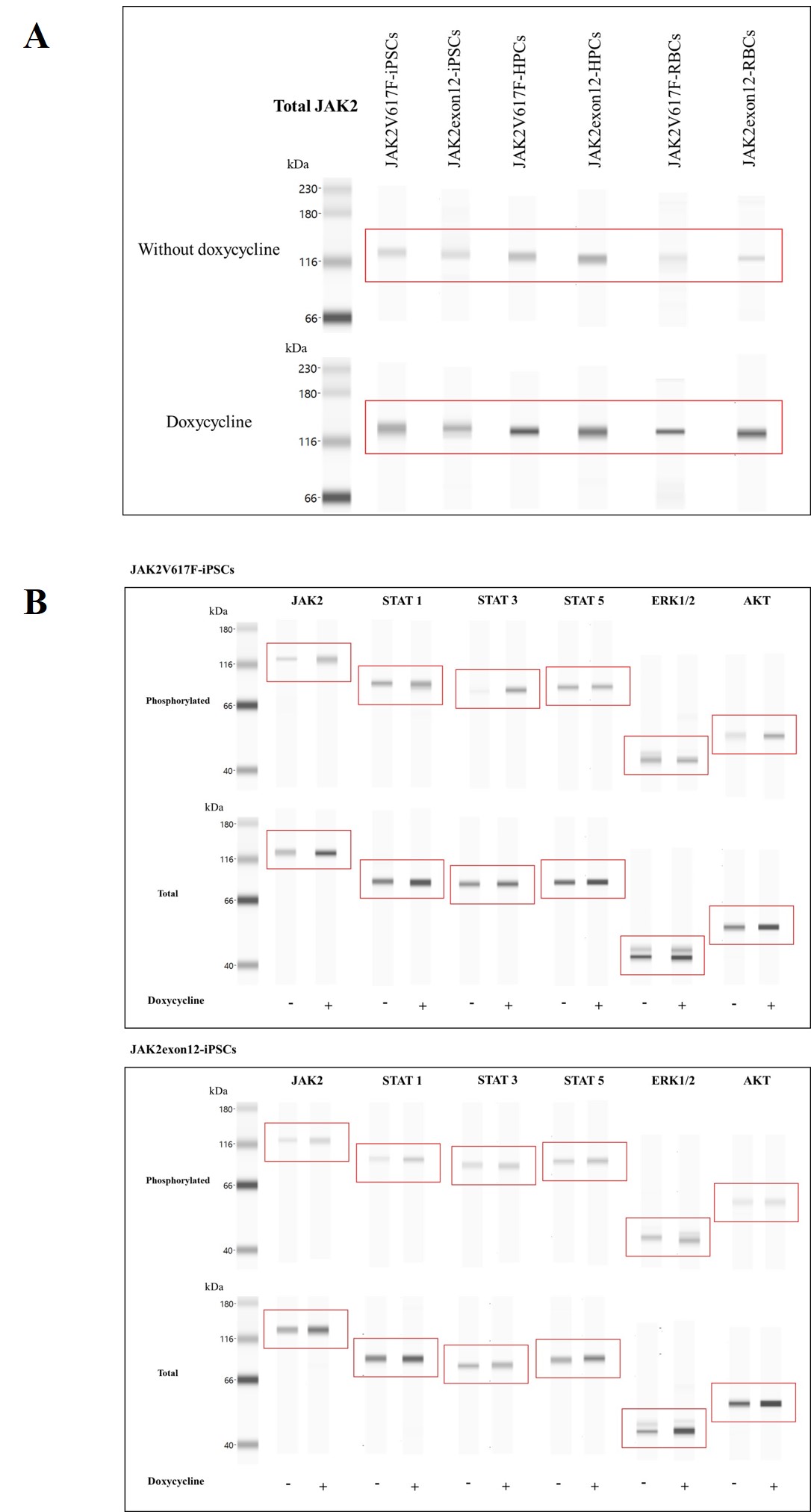


**Supplementary Figure S2.** The full-length capillary Western immunoassay. The hematopoietic progenitor cells were transferred onto fresh irradiated C3H10T1/2 cells and then cultured with a hematopoietic cell differentiation medium supplemented with 50 ng/ml thrombopoietin (TPO), 50 ng/ml stem cell factor (SCF) and 5 IU/ml erythropoietin (EPO) for 24 hours. Signaling protein analyses were performed on a capillary Western immunoassay system. Each band was electrophoresed in a separate capillary tube. A) The capillary Western immunoassay of total JAK2 proteins from JAK2V617F-expressing cells (JAK2V617F-iPSCs) and exon 12-mutated JAK2-expressing cells (JAK2exon12-iPSCs) and B) Phosphorylated and total signaling proteins in the absence and presence of doxycycline induction in JAK2V617F-iPSCs and JAK2exon12-iPSCs.

**A**

**B**
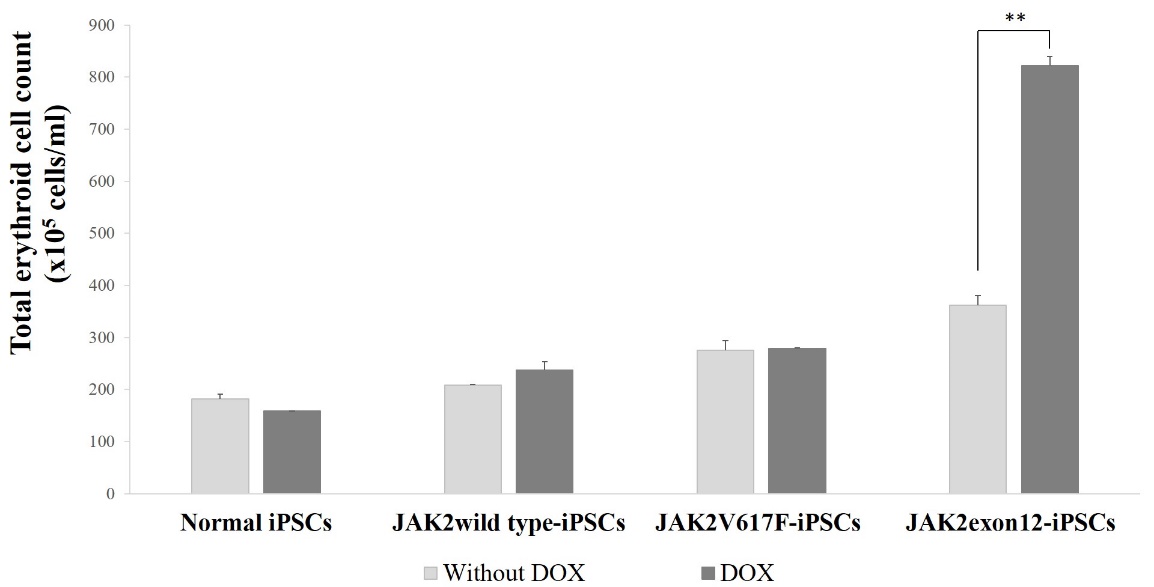


**C
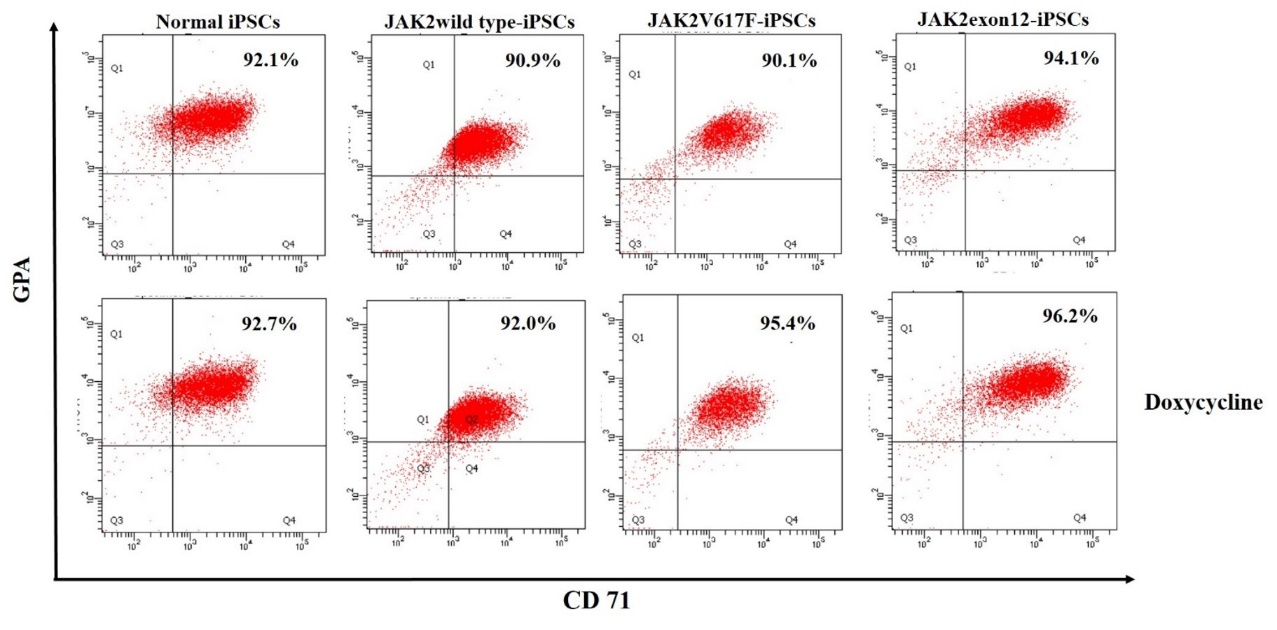
**

**
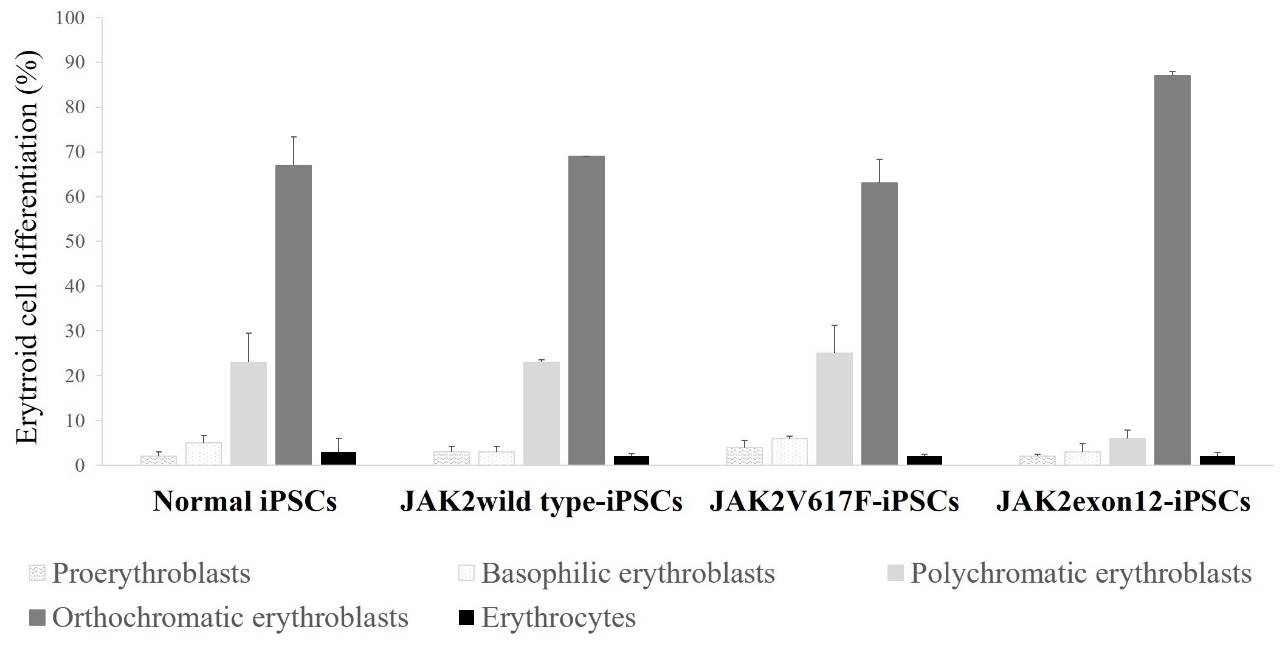
**

**Supplementary Figure S3.** Numbers and differentiation stages of erythroid cells derived from original iPSCs, iPSCs expressing wild-type JAK2 (JAK2wild-type-iPSCs), JAK2V617F (JAK2V617F-iPSCs) and JAK2 with an exon12 mutation (JAK2exon12-iPSCs). The cultured iPSCs on day 29 of erythroid differentiation were harvested for characterization. A) Total cell counts with and without doxycycline (DOX) induction were calculated by counting the total numbers of cells and multiplying by the percentages of CD71^+^GPA^+^ cells (B). The percentages of each erythroid differentiation stage as determined by morphology (C). The asterisks (**) denoted *p*<0.01.


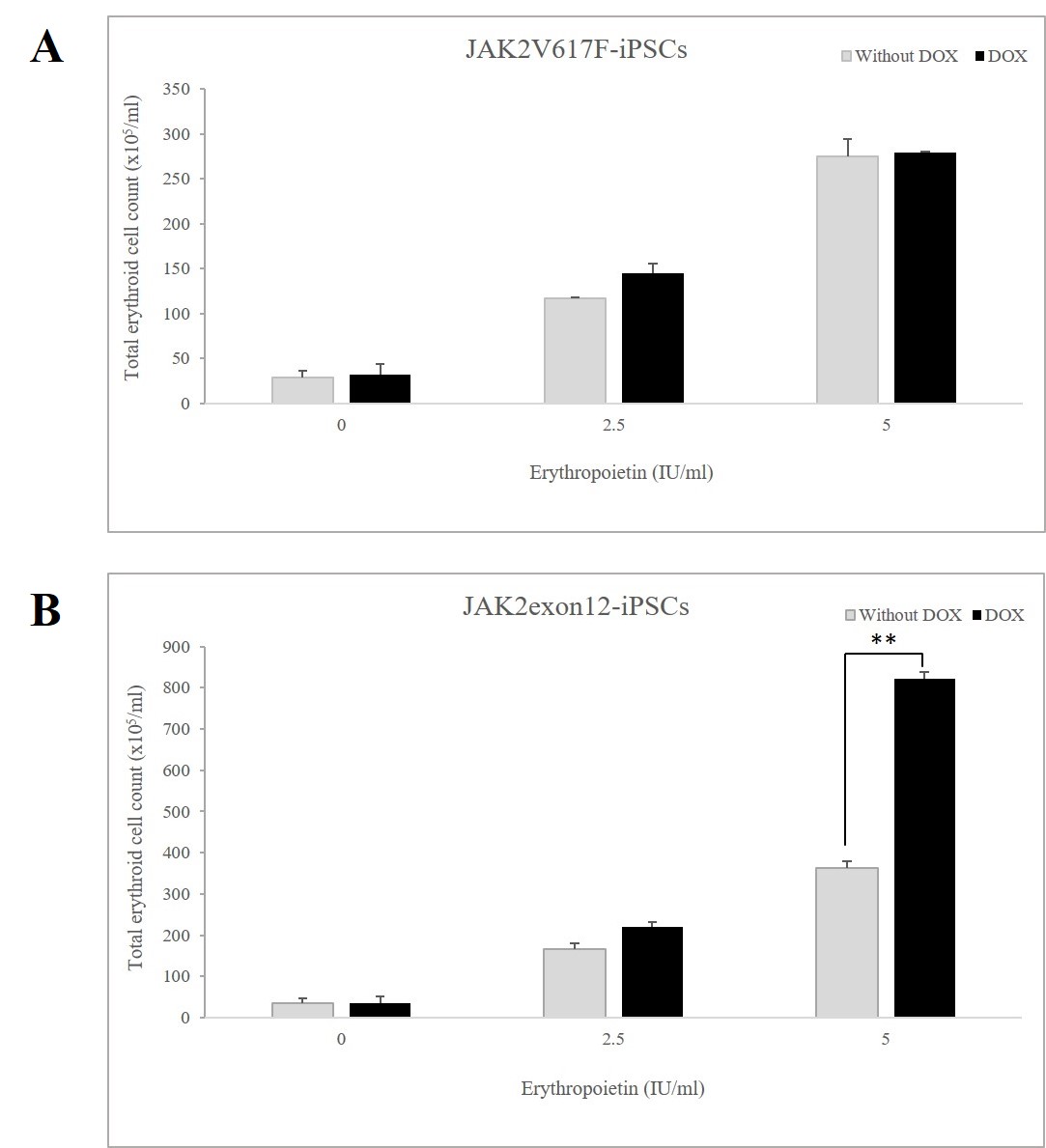


**Supplementary Figure S4.** Total erythroid cell numbers after erythroid differentiation from JAK2V617F-iPSCs (A) and JAK2exon12-iPSCs (B) in the absence (0 IU/ml) and 2.5 and 5 U/ml concentrations of erythropoietin (EPO). The hematopoietic progenitors at 5 × 10^4^ cells/ml were cultured on fresh irradiated C3H10T1/2 cells for 6 days in hematopoietic cell differentiation medium supplemented with 50 ng/ml TPO, 50 ng/ml SCF and various concentration of EPO. After 6 days, cells were transferred onto fresh irradiated C3H10T1/2 cells and cultured in hematopoietic cell differentiation medium supplemented with 0, 2.5 or 5 U/ml of only EPO for further 9 days. Non-adherent cells were analyzed on day 29 of culture. The asterisks (**) denoted *p*<0.01.


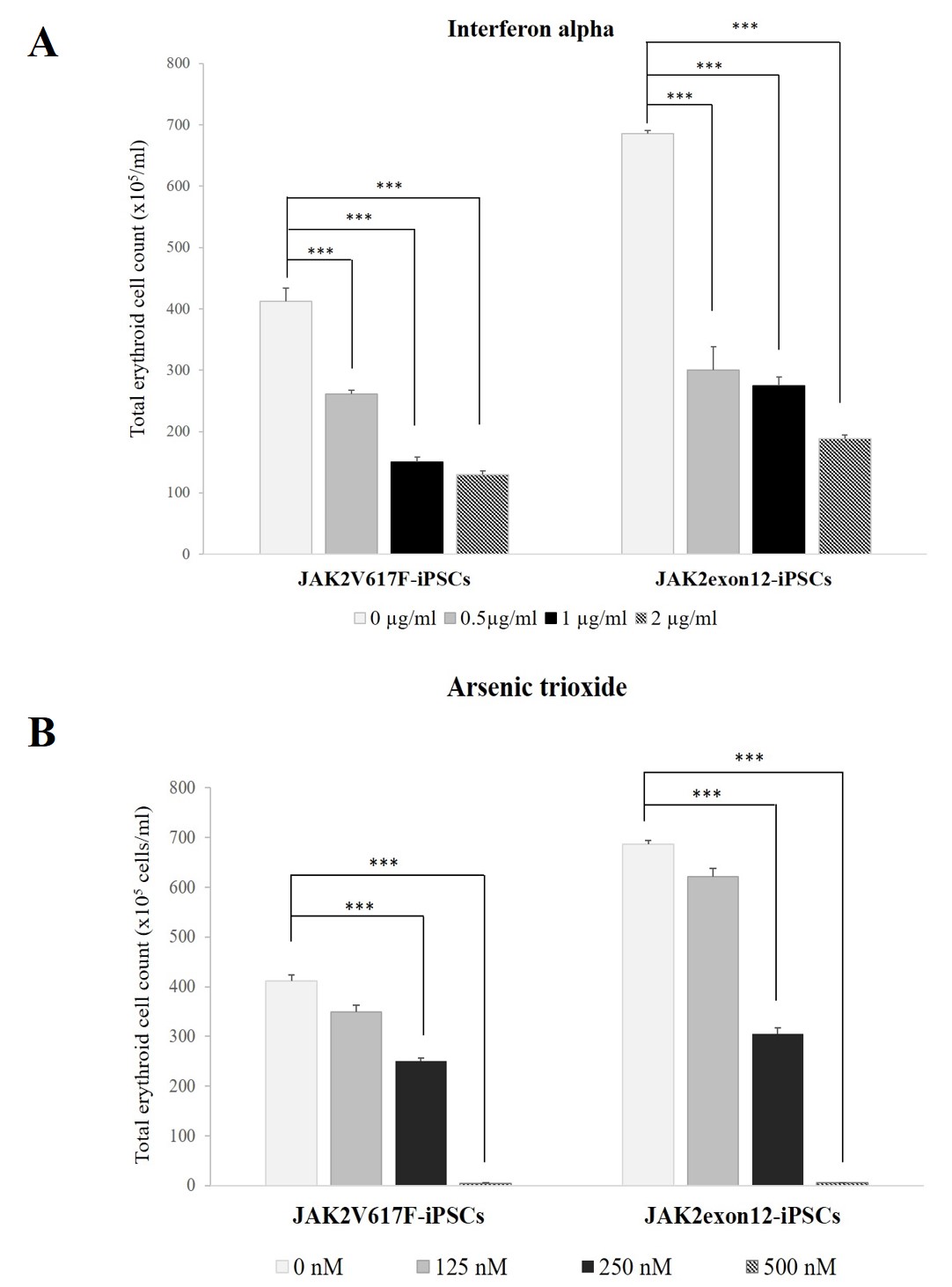


**Supplementary Figure S5.** The dose-response study of interferon alpha A) and arsenic trioxide B). The hematopoietic progenitor cells generated from modified iPSCs at 5 × 10^4^ cells/ml were differentiated into erythrocytes in the presence of interferon alpha at the concentrations of 0, 0.5, 1 and 2 µg/ml (A) or arsenic trioxide at the concentrations of 0, 125, 250 and 500 nM (B). The asterisks (***) denoted *p*<0.001.


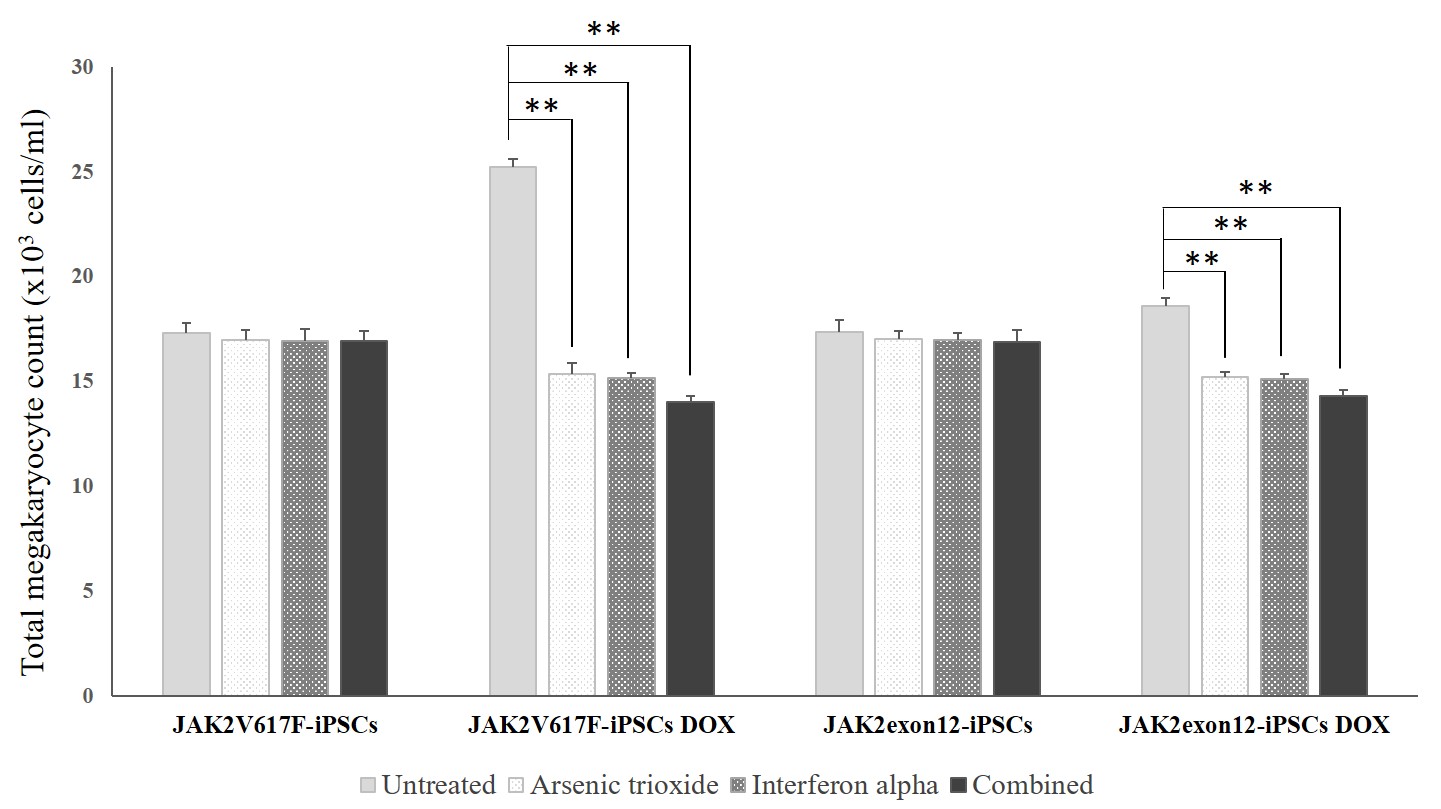


**Supplementary Figure S6.** The effects of arsenic trioxide and/or interferon alpha on megakaryopoiesis from iPSCs expressing mutant JAK2 genes: Hematopoietic progenitor cells (HPCs) at 5 × 10^4^ cells/ml from ES-Sacs (culture day 14) were differentiated into mature megakaryocytes. The HPCs were collected and passed through a 40-µM cell strainer before transferred onto inactivated OP9 cells. Cells were cultured with a hematopoietic cell differentiation medium supplemented with 100 ng/ml human TPO, 50 ng/ml human SCF, and 25 ng/ml heparin. On day 21 of culture with *vs*. without 250 nM arsenic trioxide and/or 0.5 µg/ml interferon alpha-2a, CD41-positive megakaryocyte numbers were enumerated. The experiment was performed in triplicate. In the absence of doxycycline (No mutant JAK2 expression), there was no effect of arsenic trioxide and interferon alpha. In the presence of doxycycline (DOX) induction, arsenic trioxide and/or interferon alpha showed significant inhibitory effects on these mutated JAK2 expressing cells. The asterisks (**) denoted *p*<0.01.

**
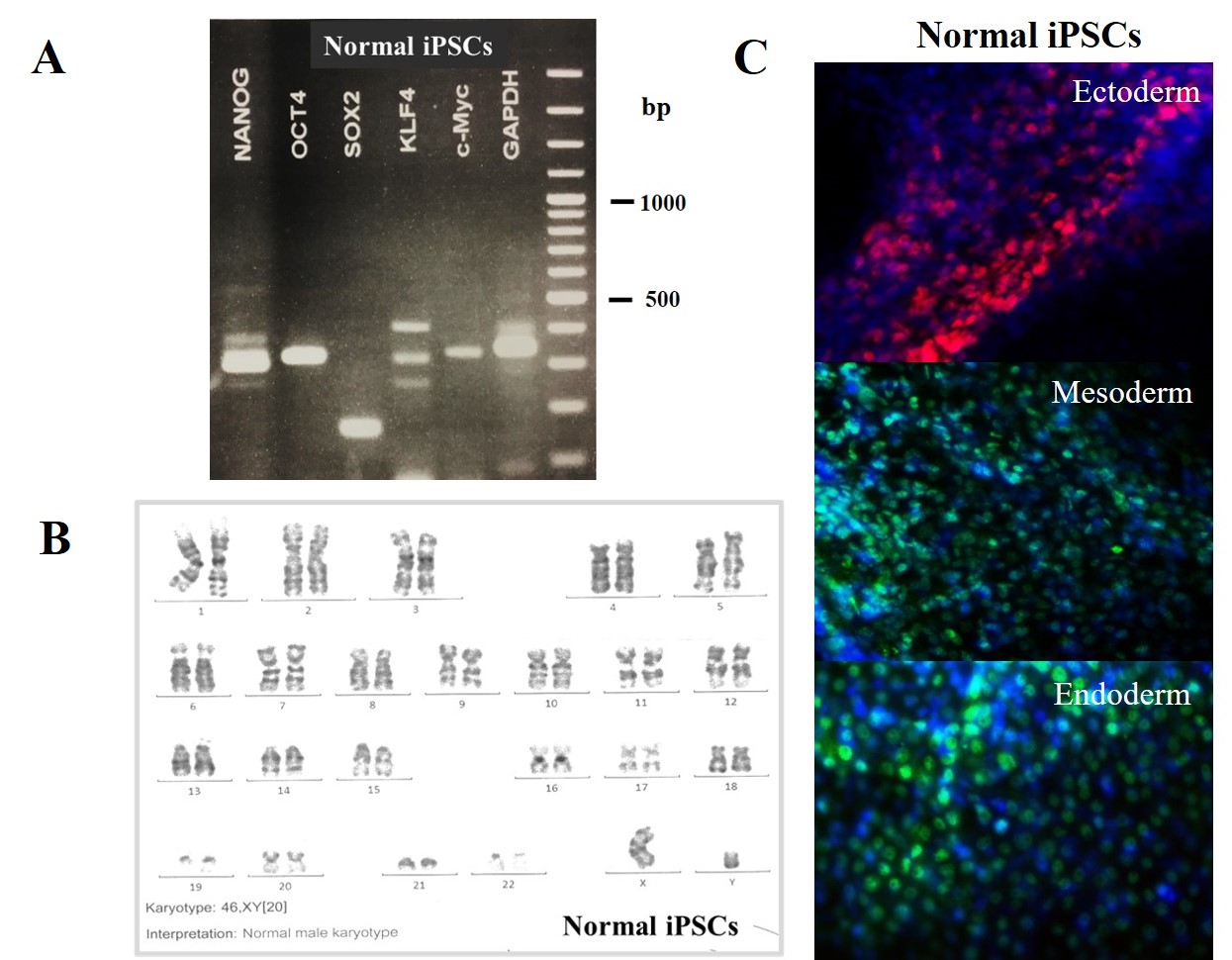
**

**Supplementary Figure S7.** Characterization of normal induced pluripotent stem cells (iPSCs) that were used in this study. The harvested normal iPSCs were evaluated for the pluripotent markers including *NANOG, OCT4, SOX2, KLF4* and *MYC* using reverse-transcriptase PCR (A). Chromosomal analysis of the original iPSCs was performed by GTG-banding analysis (B). The ability of normal iPSCs to differentiate into 3 germ layers was tested via embryoid body formation. Embryoid bodies were transferred onto 0.1% gelatin coverslips and cultured for 14 days for differentiation and stained by specific antibodies to ectoderm (red and green), mesoderm (green) and endoderm (green), as well as DAPI (blue) for nuclei (400X magnification).


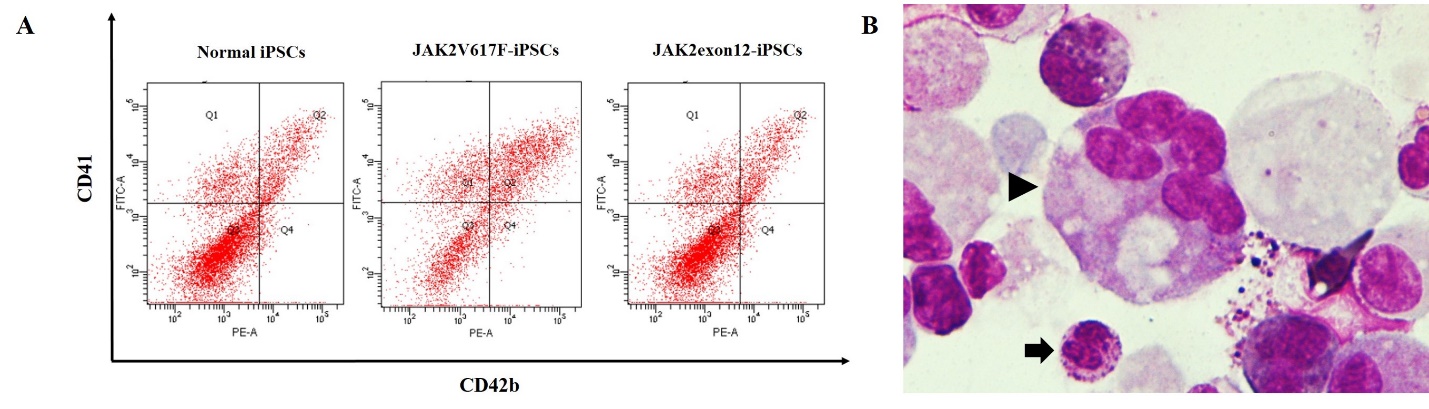


**Supplementary Figure S8.** The abilities of iPSCs in this study to differentiate into mature megakaryocytes and myeloid cells. Hematopoietic progenitor cells from ES-Sacs were collected and passed through a 40-µM cell strainer before transferred onto inactivated OP9 cells. The HPCs were cultured with a hematopoietic cell differentiation medium supplemented with 100 ng/ml human TPO, 50 ng/ml human SCF, and 25 ng/ml heparin. Megakaryocytes were harvested and analyzed on day 21 of culture with doxycycline. A) Flow cytometry for the megakaryocyte-specific markers revealed CD41+CD42b- (immature) and CD41+CD42b+ (mature) iPSC-derived megakaryocytes. B) The harvested cells were also stained by Wright-Giemsa and examined under light microscopy demonstrating a mixture of megakaryocytes (arrow head) and neutrophils (arrow).
